# Toward Hand Gesture Recognition Using a Channel-Wise Cumulative Spike Train Image-Driven Model

**DOI:** 10.34133/cbsystems.0219

**Published:** 2025-03-21

**Authors:** Yang Yu, Zeyu Zhou, Yang Xu, Chen Chen, Weichao Guo, Xinjun Sheng

**Affiliations:** ^1^ Meta Robotics Institute, Shanghai Jiao Tong University, Shanghai 200240, China.; ^2^ State Key Laboratory of Mechanical System and Vibration, Shanghai Jiao Tong University, Shanghai 200240, China.

## Abstract

Recognizing hand gestures from neural control signals is essential for natural human–machine interaction, which is extensively applied to prosthesis control and rehabilitation. However, establishing associations between the neural control signals of motor units and gestures remains an open question. Here, we propose a channel-wise cumulative spike train (cw-CST) image-driven model (cwCST-CNN) for hand gesture recognition, leveraging the spatial activation patterns of motor unit firings to distinguish motor intentions. Specifically, the cw-CSTs of motor units were decomposed from high-density surface electromyography using a spatial spike detection algorithm and were further reconstructed into images according to their spatial recording positions. Then, the resultant cwCST-images were fed into a customized convolutional neural network to recognize gestures. Additionally, we conducted an experiment involving 10 gestures and 10 subjects and compared the proposed method with 2 root-mean-square (RMS)-based approaches and a cw-CST-based approach, namely, RMS-image-driven convolutional neural network classification model, RMS feature with linear discrimination analysis classifier, and cw-CST discharge rate feature with linear discrimination analysis classifier. The results demonstrated that cwCST-CNN outperformed the other 3 methods with a higher classification accuracy of 96.92% ± 1.77%. Moreover, analysis of cw-CST and RMS features showed that the former had better separability across gestures and consistency considering training and testing datasets. This study provides a new solution and enhances the accuracy of gesture recognition using neural drive signals in human–machine interaction.

## Introduction

Hand gesture recognition plays an important role in intelligent human–machine interfaces (HMIs), such as dexterous prosthesis control, motor function rehabilitation, and mixed-reality interaction [1]. Various modalities, including electromyography (EMG), forcemyography, mechanomyography, ultrasound, and near-infrared spectroscopy, are utilized to achieve high-performance gesture recognition [2]. Among these source signals, surface EMG (sEMG) has gained much attention in the past decades owing to its noninvasiveness, wearability, and direct connections with neural control signals [3]. In this scheme, sEMG signals corresponding to different gestures are recognized as a series of patterns, in which time- or frequency-domain features are extracted and classified into gestures with machine learning algorithms [4]. To improve classification accuracy, several studies focus on exploiting more representative features [5] and designing more elaborate classifiers [6]. Afterward, more attention is paid to enhancing recognition robustness against electrode shifts [7] and adaptation ability [8]. For instance, Gao et al. [9] proposed a pattern recognition scheme using a Siamese autoencoder network to learn robust features against electrode shifts and loosening. Additionally, adaptation across users for gesture recognition was investigated through a novel spatial–temporal graph convolutional network [10]. However, the sEMG signals of these studies are merely rough and superficial counterparts of the underlying neural control signals and susceptible to the interference of noises [11,12]. To overcome this limitation, it is necessary to build a direct interface associated with the neural control signals of motor units (MUs).

Recent advances in decomposing the firing activities of MUs from high-density sEMG (HD-sEMG) signals have enabled the construction of a neural interface noninvasively. Considering these techniques, blind source separation algorithms based on convolution kernel compensation [13,14] or fast independent component analysis [15,16] are the most popular ones. In these algorithms, each MU is considered as a firing source whose discharge timings and responsive potentials are modeled in a convolutive mixture process. The motor unit spike trains (MUSTs) are estimated through compensating an unknown mixing matrix and calculating spike trains with separation vectors iteratively. Furthermore, a spatial spike detection algorithm is implemented on the whitened sEMG signals to decompose MUSTs by considering the spatial propagation characteristics of motor unit action potential (MUAP) [17]. From the perspective of the principles of neural control, the central nervous system controls the MU recruitment with common synaptic input rather than controlling each individual MU. The recruited MUs can be clustered into several functional groups based on the received common inputs [18]. Therefore, the neural control signals are from the low-dimensional MU groups, such that it is not necessary to decompose the accurate firing activities of each MU. In addition, the rigorous hypotheses of blind source separation algorithms restrict the decomposition integrity, in which some MUs’ firings are eliminated, resulting in the missing of neural control information to some extent [19]. Alternatively, the cumulative spike trains (CSTs) of a group of MUs are directly decomposed to offer upper-hierarchy control signals [19,20].

In regard to MU-relevant gesture recognition, only a few studies have conducted investigations in this emerging field. Chen et al. [21] pooled the decomposed MUSTs into several groups corresponding to each gesture, and the group with the highest activation level was recognized as the current performed gesture. To address the neural decoding across gestures in real time, a motion-wise decomposition algorithm was proposed to trace MU discharges, through which the MU-based features were extracted to train the classifier [22]. Moreover, a neuromorphic framework based on a convolutional spiking neural network was proposed to exploit the spiking encoding of the nervous system for gesture recognition, aiming to improve calculation efficiency [23]. Additionally, the firing rates of different types of decoded MUs were leveraged to recognize gestures with only 2 recording modules combined with mechanomyography, near-infrared spectroscopy, and time-domain features of sEMG [24]. Moreover, to improve the robustness of myoelectric gesture recognition against electrode shifts, the spatial information of individual MU was leveraged as prior knowledge to calibrate the shift [25].

**Fig. 1.**
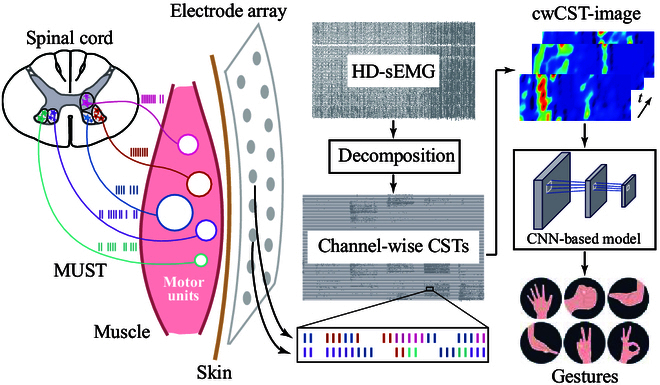
The schematic of the proposed channel-wise cumulative spike train image (cwCST-image)-based gesture recognition method. The recorded high-density surface electromyography (HD-sEMG) signals are decomposed into channel-wise cumulative spike trains (CSTs), transformed into a cwCST-image according to the channel distribution and decoded to different gestures by a convolutional neural network (CNN)-based model. The CSTs in each channel comprise the discharges of motor units adjacent to the corresponding electrode, represented by bars and circles in different colors. MUST, motor unit spike train.

It is noteworthy that the aforementioned studies are still focused on the temporal or frequency characteristics of MUs while ignoring the spatial information of neural signals. In physiology, the generation of movements relies on the synergy of muscles distributed in different locations, which receive neural drives across certain MU groups [18]. As such, these movements should have particular spatial representations with respect to MUs. The quantification of the spatial activation patterns of MUs in finger muscles shows that these patterns are distinct for finger movements [26]. Similarly, a semisupervised finger recognition scheme also validates the differences of spatial MU activation [27]. Inspired by these facts, we propose to extract valuable representations from the spatial activation of low-dimensional neural control signals (CSTs) and build associations between these representations and gestures for neural interfacing. Specifically, we present a channel-wise CST image (cwCST-image)-driven model for hand gesture recognition (Fig. 1), in which channel-wise cumulative spike trains (cw-CSTs) are decomposed from HD-sEMG and reconstructed into cwCST-images based on the spatial location of recording channels. Moreover, the representations of cwCST-images are extracted by a customized convolutional neural network (CNN) and further mapped into hand gestures. The contributions of this study can be concluded as follows: (a) The proposed method builds connections between gestures and direct low-dimensional neural control information (i.e., channel-wise CST); (b) The spatial information underlying the activated MUs in different area are introduced for enhancing the performance of hand gesture recognition.

## Materials and Methods

### Subjects

We recruited 10 subjects (S1 to S10, 8 males and 2 females, 23.8 ± 2.3 years) to participate in the experiments without reporting any neuromuscular disorders or movement diseases. The whole procedures were relayed to the participants in advance and conformed to the Declaration of Helsinki. The experimental protocols were approved by the Institutional Review Board of Human Research Protection of Shanghai Jiao Tong University (Approval No. B2020026I).

### Experiments

#### Data recording

**Fig. 2.**
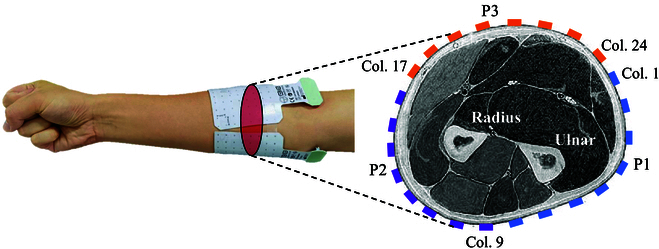
The electrode distribution in the experiment. Three pieces of 8 × 8 electrodes (P1 to P3) are attached around the forearm, and the location with respect to muscles is depicted. Col., column.

The HD-sEMG signals were recorded with 3 pieces of 8 × 8 electrode arrays (GR10MM0808, OT Bioelettronica, Italy) with a 10-mm inter-electrode distance and a 3-mm electrode diameter. These arrays were uniformly distributed around the forearm from the radius to the ulna (Fig. 2), attached on one-third length of the forearm to the elbow, while the reference electrode was enwound on the wrist. Prior to data acquisition, the skin of each subject was cleaned with alcohol wipes and the electrode arrays were affixed with flexible double-sided adhesive tape with gel injection to enhance the conduction ability of the electrode–skin interface. The signals were amplified with a gain of 500 and sampled with 2,048 Hz using a commercial acquisition device (EMG-USB2+, OT Bioelettronica, Italy).

#### Experimental protocol

**Fig. 3.**
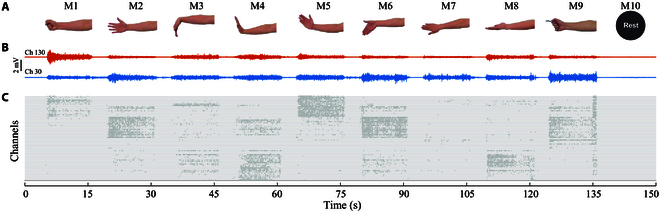
Experimental setups and representative results in a trial. (A) Hand gestures involved in the experiment. (B) sEMG signals of 2 selected channels. (C) Decomposed cw-CSTs of a piece of the electrode array (64 channels). Ch, channel.

During the experiment, subjects sat in a chair comfortably and were instructed to perform 10 discrete gestures including rest (M1 to M10, Fig. 3A) in sequence with their dominant hand. Those motions comprised hand grasp, hand open, wrist flexion, wrist extension, wrist abduction, wrist adduction, wrist pronation, wrist supination, key pinch, and rest. Each motion was sustained for 10 s, and there was a 5-s rest period between 2 motions, resulting in each trial lasting 150 s. The order of M1 to M9 occurred in the experiment was random, while the rest state was performed as the last one. For each subject, the trial was repeated 8 times and a rest period of 1 to 2 min was provided between trials to avoid muscle fatigue. Moreover, the activation level of each motion was expected to be similar across trials, before which we provided subjects with sufficient time to familiarize themselves with the protocols. The corresponding sEMG signals of S1 in a trial are depicted in Fig. 3B.

### Channel-wise CST image-driven model

**Fig. 4.**
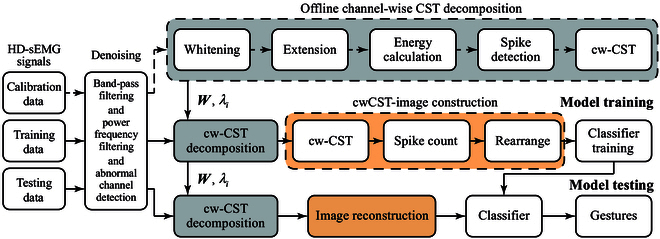
Data processing of the cwCST-image-based gesture recognition method. The recorded HD-sEMG signals are preprocessed to eliminate noises by filtering and removal of the abnormal channels. Among these signals, calibration data are utilized to determine the parameters, W and $\lambda_{i}$, of the cw-CST decomposition algorithm. Subsequently, training data are decomposed by a spatial spike detection algorithm to cw-CSTs, which are transformed into images and used to train the CNN model-based classifier. Additionally, the testing data are decomposed, transformed, and recognized as gestures.

The overview of data processing pipeline is illustrated in Fig. 4, including the preprocessing of signals on denoising, offline calibration of decomposition parameters, training of the gesture recognition model, and evaluation of the performance of the model in a testing dataset. The detailed information are as follows.

#### Data preprocessing

The recorded sEMG signals were divided into calibration data, training data, and testing data. All these signals were denoised in the same way to eliminate the adverse impact of low-frequency movement artifacts, power-line interference, and high-frequency noises. To this end, the signals were band-pass-filtered with 20 to 450 Hz and then processed using a notching-comb filter with a cutoff frequency of 50 Hz and its multiples. Moreover, we implemented channel selection [19] to remove the abnormal ones so as to improve the decomposition accuracy of channel-wise CSTs.

#### cw-CST decomposition and image reconstruction

To extract the cumulative firing activities of MUs, we adapted and improved a CST estimation algorithm based on the spatial propagation characteristics of MUAP, in which the discharge timings of MUs could be decomposed from the HD-sEMG signals using spatial spike detection [19]. It is noteworthy that the HD-sEMG signals from all of 3 electrode arrays (192 channels) were leveraged during decomposition. Here, a brief introduction on the decomposition algorithm is given below, including data whitening and extension, energy calculation, and spike detection.

The multichannel sEMG signals can be modeled as a convolutive mixture model in both the temporal and spatial domains, as shown in Eq. (1):

$$X\left(t\right)=\mathrm{HS}\left(t\right)+\omega \left(t\right)$$ (1)

with

$$X\left(t\right)={\left[x_{1}\left(t\right),\ldots ,x_{M}\left(t\right)\right]}^{T}$$ (2)

$$S\left(t\right)={\left[s_{1}\left(t\right),\ldots ,s_{M}\left(t\right)\right]}^{T}$$ (3)

$$\omega \left(t\right)={\left[\omega_{1}\left(t\right),\ldots ,\omega_{M}\left(t\right)\right]}^{T}$$ (4)

H is an M×N mixing matrix composed of the corresponding responsive action potentials across all channels, where *M* and *N* are the numbers of recording channels and MUs, respectively. The responsive action potential of the *j*th MU in the *i*th channel is denoted as $h_{\mathrm{ij}}=\left[h_{\mathrm{ij}}\left(0\right),\ldots ,h_{\mathrm{ij}}\left(L-1\right)\right]$, where *L* is the length of the MU’s response. $S\left(t\right)$ represents the discharge timings of MUs, while $\omega \left(t\right)$ is the noise.

(a) Data whitening and extension: The high correlation between multichannel EMG signals makes it difficult to separate the unique discharge information of MUs. To overcome this limitation, we firstly performed whitening transformation based on zero-phase component analysis [28] to express the signals in Mahalanobis space. The whitened signals $Z\left(t\right)$ are presented as

$$Z\left(t\right)=\mathrm{WX}\left(t\right)$$ (5)

The whitening matrix W of zero-phase component analysis transformation can be calculated as

$$W=\Sigma^{-\frac{1}{2}}=U\Lambda^{-\frac{1}{2}}U^{T}$$ (6)

where $\Sigma =E\left({\mathrm{XX}}^{T}\right)$ represents the covariance matrix of sEMG signals X, and the operator $E\left(\cdot \right)$ denotes the mathematical expectations. Eigendecomposition can be utilized to obtain U and Λ as follows:

$$\Sigma =U\Lambda U^{T}$$ (7)

To enhance the calculation efficiency of matrix operation, we extended the whitened signals $Z\left(t\right)$ into an *R*-delayed version: $\begin{array}{c} \bar{Z}\left(t\right)={\left[{\bar{z}}_{1_{1}}\left(t\right),\ldots ,\;{\bar{z}}_{1_{r}}\left(t\right),\ldots ,\;{\bar{z}}_{M_{1}}\left(t\right),\ldots ,\;{\bar{z}}_{M_{r}}\left(t\right)\right]}^{T}=\left[z_{1}\left(t\right),\ldots ,\right. \end{array}$$\begin{array}{c} {\left.z_{1}\left(t-R+1\right),\ldots ,z_{M}\left(t\right),\ldots ,z_{M}\left(t-R+1\right)\right]}^{T} \end{array}$.

(b) Energy calculation: Next, the energy of the *i*th channel is calculated as the sum of squares regarding the adjacent *R* samples:

$$e_{i}\left(t\right)=\sum_{r=1}^{R}{\bar{z}}_{i_{r}}^{2}\left(t\right)$$ (8)

where ${\bar{z}}_{i_{r}}$ indicates the *r*th delayed sample of $\bar{Z}\left(t\right)$ in the *i*th channel. Subsequently, the firing activities of relevant MUs in the *i*th channel is expected to be decomposed from $e_{i}\left(t\right)$.

(c) Spike detection: The spatial propagation characteristics of MUAP shows that it conducts along the direction of muscle fibers with little attenuation and with quick amplitude attenuation in the direction perpendicular to the fibers. Considering this property, there should exist a peak in a local spatial area at the MU discharge instant. Consequently, we propose a spatial spike detection method to identify the discharge timings of MUs belonging to a specific channel. Specifically, a spatial sliding window is utilized to identify the possible discharges in a subregion of the electrode array through scanning the entire electrode. The size of the sliding window in spatial and temporal dimensions is 3 × 3 and 5 ms, respectively. An MU spike at time instant *t* should satisfy 2 conditions: (a) the energy of the *i*th channel $e_{i}\left(t\right)$ in Eq. (8) exceeds a predefined threshold $\lambda_{i}$ and (b) the amplitude of the candidate spike has the largest value among the channels of the spatial sliding window as well as in the temporal dimension. The threshold $\lambda_{i}$ was calculated through a *K*-means clustering algorithm using the one-trial calibration data in Fig. 4. Specifically, we clustered the candidate spikes into activated and inactivated ones using a clustering evaluation metric silhouette coefficient equal to 0.7 [29]. Accordingly, the detection threshold $\lambda_{i}$ is denoted as the mean value of 2 clustering centroids:

$$\lambda_{i}=\text{mean}\left(e_{i,\mathrm{low}},\;e_{i,\text{high}}\right)$$ (9)

where $e_{i,\mathrm{low}}$ and $e_{i,\text{high}}$ represent the centroids of 2 clusters, respectively. The decomposed CSTs of all channels can be expressed as

$$\hat{S}\left(t\right)={\left[{\hat{s}}_{1}\left(t\right),\ldots ,\;{\hat{s}}_{M}\left(t\right)\right]}^{T}$$ (10)

where ${\hat{s}}_{i}\left(t\right)=1$ and ${\hat{s}}_{i}\left(t\right)=0$ denote the discharge and nondischarge samples of the *i*th channel, respectively.

(d) cwCST-image reconstruction: Next, we calculated the discharge rate of spike trains in each channel, ${\hat{s}}_{i}$, using a 200-ms sliding window with a 50-ms increment. To characterize the spatial patterns of the cumulative neural information of MUs, we transformed the resultant discharge rate of 192-channel cw-CSTs into an 8 × 24 array according to the electrodes’ spatial distribution, thus obtaining an instantaneous image at every sampling instant, namely, the cwCST-image.

#### cwCST-CNN model architecture

**Fig. 5.**
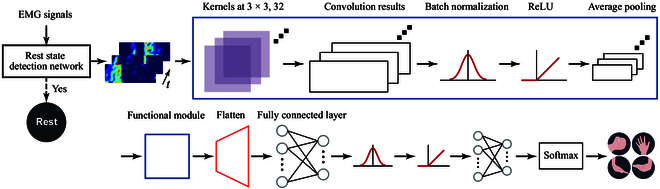
The architecture of the proposed method based on the CNN model and cwCST-image. Prior to classifying cwCST-images into gestures, a rest state detection network is firstly adopted to distinguish the rest state from other gestures. Then, the constructed cwCST-images are fed into the CNN model. The detailed layers of the model are presented. EMG, electromyography; ReLU, rectified linear unit.

The architecture of the proposed cwCST-image-driven model is illustrated in Fig. 5, including the rest state detection network and cwCST-image-driven hand gesture recognition network. In this study, we term this model as “cwCST-CNN”. The rest state detection network is a CNN-based network composed of 2 cascaded modules of a 2-dimensional convolutional layer, a batch normalization (BN) layer, a rectified linear unit, an average pooling layer, 2 fully connected layers, and a LogSoftmax layer. The input of this network is an 8 × 24 × 1 image constructed from the normalized root-mean-square (RMS) features of all channels, while the output is rest or nonrest state. When a nonrest state is recognized, the cwCST-images (8 × 24 × 1) are then fed into the gesture recognition network. Similarly, it has 15 layers, including 2 cascaded functional modules, a flatten layer, fully connected layers, a BN layer, a rectified linear unit, and a LogSoftmax layer. For convolution layers in those functional modules, the kernels are both with a size of 3 × 3, striding one and same-padded, and are with numbers of 32 and 16, respectively. Two average pooling layers in the modules are with 2 × 2 kernels, striding one, and zero-padded. Additionally, a BN layer is utilized to avoid overfitting. For detailed information on the network architecture, refer to Fig. 5.

#### Model training and testing

A randomly selected trial was used to calibrate the parameters (W and $\lambda_{i}$) of the cw-CST decomposition model, while the remaining trials were leveraged to evaluate the gesture recognition model as training and testing data. This configuration enabled the prospective requirements for online decomposition of cw-CSTs and gesture classification. To investigate the impact of training trials on the classification accuracy, we trained the classification model with *k* (1≤k≤6) trials, while the rest data were used to test the performance. For the training of the cwCST-CNN model, we used the adaptive moment estimation (Adam) optimizer with the entire data of training trials for training and validation. The minibatch size was selected as 1,000, and the epoch number was set to 100 empirically. In addition, the learning rate was set to 0.01 initially and automatically changed by the Adam optimizer. Moreover, we used a negative log-likelihood loss function to train the model and determined the final parameters of the cwCST-CNN model when optimal classification accuracy was obtained during validation.

#### Comparative gesture recognition methods

To validate the effectiveness of cw-CST-based features and the superiority of introducing spatial information, we compared the proposed cwCST-CNN with 3 approaches, i.e., RMS-image-driven CNN classification model (RMS-CNN), cw-CST discharge rate feature with linear discrimination analysis (LDA) classifier (cwCST-LDA), and RMS feature with LDA classifier (RMS-LDA). RMS has been a popular feature and regarded as a benchmark in the field of myoelectric control [30,31], while the performance of LDA classifiers has been demonstrated to be comparable to that of other more complicated classifiers [22,32]. Specifically, for the discharge rate of cw-CST, we set a threshold to limit the maximum values to avoid the paradoxical firings of MUs, while normalization across trials was conducted with regard to RMS feature. Moreover, the architecture of RMS-CNN was the same as that of cwCST-CNN. For cwCST-LDA and RMS-LDA, the input is a feature vector of 192 and the feature was obtained by a 200-ms sliding window with a 50-ms increment.

### Performance evaluation metrics

We evaluated the gesture recognition methods considering classification accuracy, in which the ratio between the number of correctly classified gestures and the total number of predicted gestures was adopted. Additionally, quantification of the feature distributions of cw-CST and RMS was also implemented with the separability index (SI) and repeatability index (RI) [24]. SI measures the diversity of different gestures features, while RI describes the consistency of features between training and testing data for a specific gesture. A larger SI denotes greater interclass distance in feature space and thus better separability of different gestures. Similarly, RI has a negative correlation between the consistency of testing and training data.

### Statistical analysis

To compare the classification accuracy of different methods, including cwCST-CNN, RMS-CNN, cwCST-LDA, and RMS-LDA, we analyzed the statistical significance between the accuracy differences of these methods using one-way analysis of variance in SPSS (SPSS Statistics 26, IBM, USA). Similarly, statistical analysis of the SI and RI of the cw-CST and RMS features was conducted in the same way. Prior to the analysis, the homogeneity of variance and normal distribution of the evaluation metrics were checked, and then multiple comparison analysis with the Bonferroni approach was conducted. The significance level was set to 0.05.

## Results

### Activation patterns of cwCST-images

**Fig. 6.**
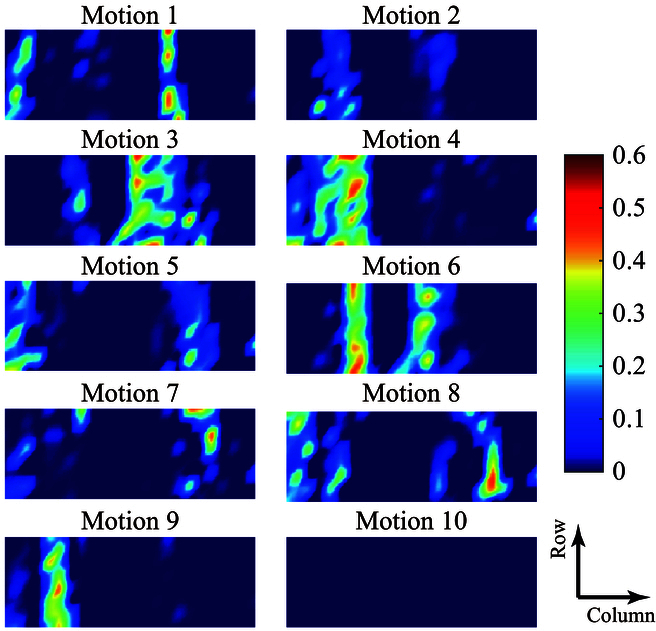
The activation patterns inside cwCST-images across gestures (motions 1 to 10) of S1. Motion 10 corresponds to the rest state, while other motions denote those gestures in Fig. 3 accordingly. A warmer tone indicates a higher activation level.

In this study, we made qualitative analysis of the patterns of the cwCST-images of different gestures, aiming to show the differentiated activation patterns underlying the neural innervation of MUs. Fig. 6 shows the unique activation patterns of cwCST-images for each gesture (M1 to M10) of S1. Each image was obtained by averaging all samples across all trials, which was normalized by the maximum discharge rate. Obviously, the activation pattern of each gesture differs from those of other motions, since different gestures involve different muscles, which have physiologically variant distributions in space. As such, this correspondingly contributes to the recognition of different gestures to some extent.

### Recognition accuracy of different methods

**Fig. 7.**
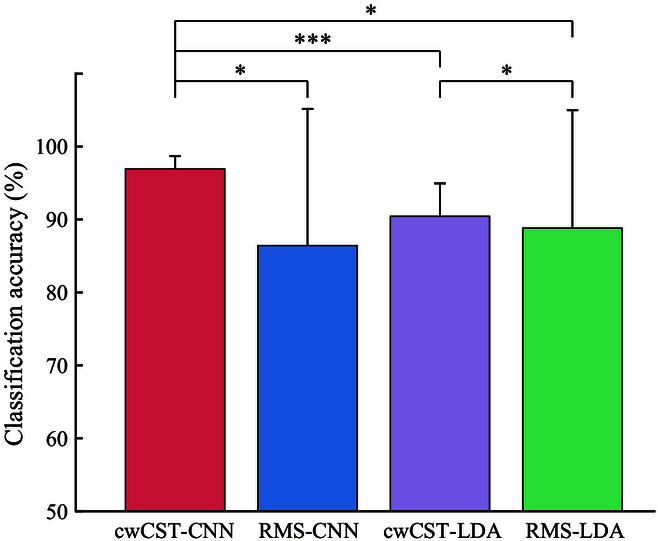
The classification accuracy of 4 comparative methods. The histogram and error bar denote corresponding mean values and standard deviation across all subjects with 6 trials for training, respectively. Asterisks represent significance levels (*P<0.05; **P<0.01; ***P<0.001). cwCST-CNN, cw-CST image-driven model; RMS-CNN, root-mean-square (RMS)-image-driven CNN classification model; cwCST-LDA, cw-CST discharge rate feature with linear discrimination analysis (LDA) classifier; RMS-LDA, RMS feature with LDA classifier.

Apart from the qualitative analysis, we also calculated the average classification accuracy of different methods across subjects with respect to all gestures (Fig. 7). The average classification accuracies of cwCST-CNN, RMS-CNN, cwCST-LDA, and RMS-LDA are 96.92% ± 1.77%, 86.42% ± 18.73%, 90.44% ± 4.50%, and 88.83% ± 16.14%, respectively. Note that the results in Fig. 7 are with 6 trials for training. Statistical analysis shows that there are significant differences between cwCST-CNN and RMS-CNN (P<0.05), cwCST-LDA (P<0.001), and RMS-LDA (P<0.05). Furthermore, significant difference exists in terms of cwCST-LDA and RMS-LDA with P<0.05.

**Fig. 8.**
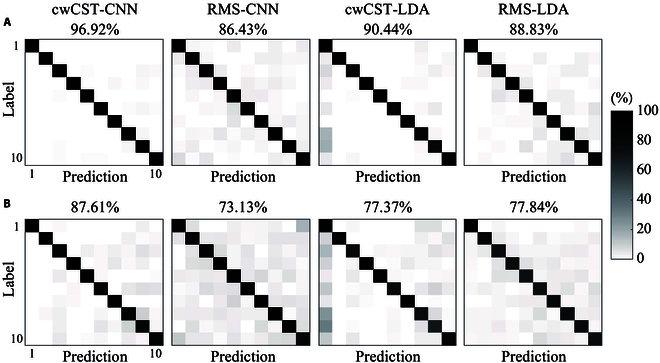
The confusion matrices of different methods based on cw-CST and RMS across all subjects. (A) and (B) are the results of those trained with 6 trials and 1 trial, respectively. The horizontal axis denotes the predicted gestures, while the vertical axis represents the ground truth labels.

Fig. 8A and B present the confusion matrices of the classification accuracy of the 4 comparative methods with 6 trials and 1 trial for training, respectively. The average classification accuracy in Fig. 8A is the same as that in Fig. 7, while those of cwCST-CNN, RMS-CNN, cwCST-LDA, and RMS-LDA in Fig. 8B are 87.61% ± 6.12%, 73.13% ± 3.88%, 77.37% ± 8.99%, and 77.84% ± 6.40%, respectively. Compared with RMS feature, cwCST-based methods achieve a higher accuracy in both of the 2 conditions when the same classification method is adopted. Additionally, the interclass misclassification is much more serious for the 2 RMS-based methods, with more darker areas considering the off-diagonal elements.

### Effect of training trials on recognition accuracy

**Fig. 9.**
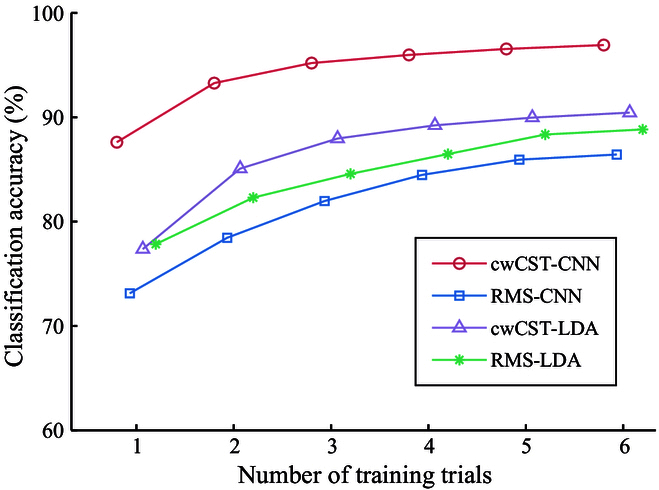
The relations between classification accuracy and the number of training trials of different approaches. The lines with circles, squares, triangles, and asterisks denote cwCST-CNN, RMS-CNN, cwCST-LDA, and RMS-LDA, respectively.

The impact of the number of training trials on the classification accuracy is depicted in Fig. 9. As shown in Fig. 9, the classification accuracy increases with the involvement of more training trials for all methods and gradually becomes steady when exceeding 4 trials. Furthermore, no matter how many training trials are used, the classification accuracy of cwCST-CNN is always higher than those of other 3 methods. Despite only one trial leveraged for training, cwCST-CNN is still able to obtain a classification accuracy of more than 85%. This reveals that perhaps the proposed cwCST-CNN is much superior in generalization compared with other 3 approaches and just requires less data to adapt to different circumstances.

## Discussion

### Superiority of the proposed cwCST-CNN model

In this study, we decomposed the direct control information of neural system (CSTs) from HD-sEMG signals and transformed them into cwCST-images according to their spatial distributions. Combined with a CNN model, the underlying neural control information of cwCST-images can be extracted effectively in both local and global areas through the spatial convolution of kernels. As a classic feature in the field of myoelectric control, RMS was used as a benchmark to validate the effectiveness and superiority of the proposed method. On the one hand, we constructed RMS-images and fed them into the CNN model to compare the difference between neural control information and confounding control sources. On the other hand, we also introduced an LDA classifier combined with the discharge rate of CSTs, aiming to evaluate the performance of introducing spatial information by constructing images for CNN model. The results demonstrated that the proposed cwCST-CNN had better performance than other 3 methods with a higher recognition accuracy of 96.92% ± 1.77% for the 10 gestures. The distinctions between cwCST-images and RMS-images might result from the fact that RMS is a rough estimation of the neural excitation and is susceptible to the interferences of sEMG noises [11]. Meanwhile, the decomposed cw-CST is an estimation of the direct control information, which is more robust [33,34]. As such, it is critical to establish direct connections with neural control information for HMI, and the introduction of spatial correlations between different MUs contributes to enhancing the estimation accuracy. Previous studies also provide evidence for these perspectives [26,35]. Additionally, several neural interfaces for gesture recognition have been reported in recent years. The classification accuracy of Chen et al. [21,22] exceeds 90% for recognizing more than 10 gestures. Moreover, a neuromorphic framework processing MU activities based on a spiking neural network achieves a 95% classification accuracy for 10 gestures [23]. Considering the proposed method, we recognized 10 gestures with a classification accuracy of 96.92% ± 1.77%, which is comparable with or even better than those of the aforementioned works.

### Feature analysis of channel-wise CST and RMS

**Fig. 10.**
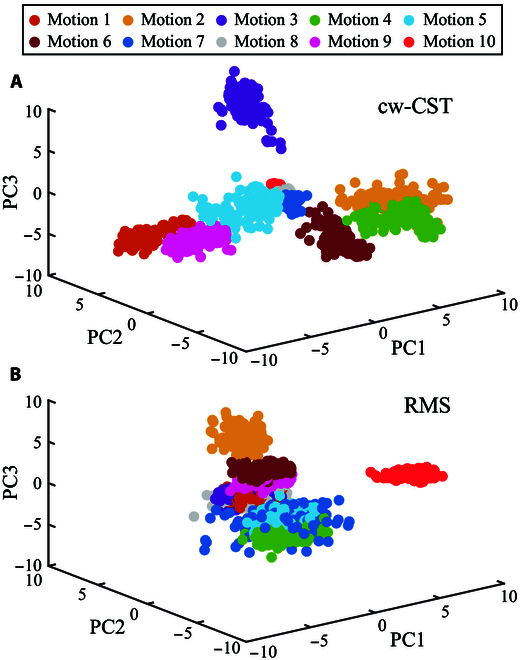
Feature distributions of different motions of a representative subject (S1). (A) and (B) represent the first 3 principal components of features extracted from cw-CST and RMS features, respectively. Different motions are denoted with dots in various colors.

**Fig. 11.**
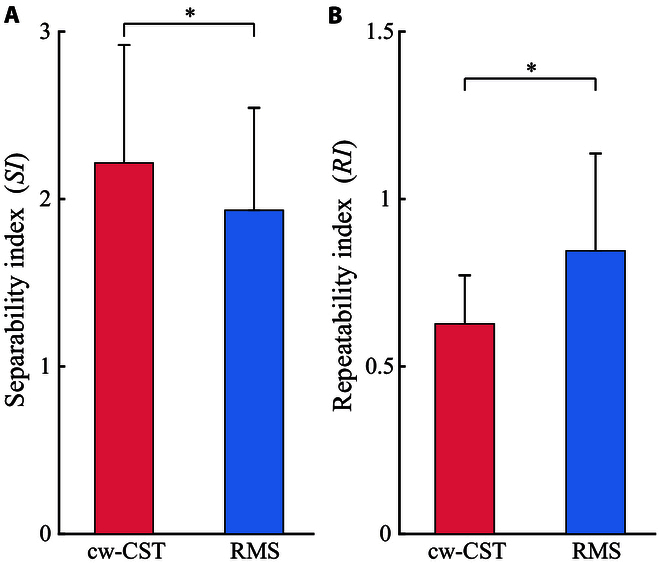
(A and B) The separability index (*SI*) and repeatability index (*RI*) of cw-CST and RMS features across subjects. Asterisks represent significance levels (*P<0.05).

To interpret the performance of these 4 gesture recognition methods, we analyzed the distribution of cw-CST and RMS in feature space qualitatively and quantitatively. Specifically, principal component (PC) analysis was respectively implemented on the cw-CST and RMS features of all gestures, and then the first 3 PCs were utilized to visualize their corresponding distributions in feature space (Fig. 10). Fig. 10A and B present the distributions of the cw-CST and RMS features of M1 to M10, respectively. It is obvious that the features of different gestures of cw-CST are more separable, compared with those of RMS features. This result accounts for the differences in the classification accuracy of cwCST- and RMS-based gesture recognition methods in Fig. 7. The greater interclass distances of all gestures are beneficial for the enhancement of classification accuracy. In particular, the PCs of RMS feature in terms of rest state (motion 10) in feature space is far away from those of other gestures. As such, we added a rest state detection network at the beginning of cwCST-CNN. Additionally, to characterize the feature distribution of cw-CST and RMS quantitatively, we calculated the SI and RI metrics of the original cw-CST and RMS features as described in the “Performance evaluation metrics” section. The Table shows the SI and RI of the cw-CST and RMS features of all subjects, while Fig. 11 presents the average values of these 2 metrics across subjects. The average SIs of cw-CST and RMS are 2.21 ± 0.70 and 1.93 ± 0.61, respectively, while the corresponding RIs of cw-CST and RMS are 0.63 ± 0.14 and 0.85 ± 0.29, respectively. Furthermore, statistical analysis shows that there are significant differences (P<0.05) between cw-CST and RMS features considering both SI and RI. As mentioned in the “Performance evaluation metrics” section, a larger SI means larger intervals between different classes and a smaller RI implies better consistency between training and testing data, which is beneficial for classifying different gestures. Hence, the larger SI and smaller RI of cw-CST could provide a reasonable explanation for the higher classification accuracy of cw-CST-based approaches.

**Table.** The separability index (SI) and repeatability index (RI) of cw-CST and RMS features

Metrics	FeatType	S1	S2	S3	S4	S5
SI	cw-CST	3.24	3.53	2.02	2.54	1.39
	RMS	2.38	2.54	2.49	2.24	2.37
RI	cw-CST	0.61	1.00	0.44	0.59	0.49
	RMS	0.66	0.60	0.55	0.68	0.67
Metrics	FeatType	S6	S7	S8	S9	S10
SI	cw-CST	1.87	1.73	1.87	2.61	1.36
	RMS	0.88	2.02	1.08	2.20	1.12
RI	cw-CST	0.56	0.69	0.56	0.66	0.69
	RMS	1.09	0.68	1.18	0.85	1.49

### Potential applications, limitations, and future work

Gesture recognition using neural signals has many potential applications, such as prosthetic control [36], stroke rehabilitation [37], and virtual input for smart devices [38]. Different from those based on contaminated sEMG signals, the proposed scheme builds direct associations between gestures and the decomposed neural signals, which might be beneficial for more robust gesture recognition. However, there are some limitations for the proposed method. For instance, the decomposition of cw-CST and the reconstruction of cwCST-images are both dependent on HD-sEMG, where a multichannel electrode array is required. In this case, it might be inconvenient for wearable devices to some extent. Fortunately, the emerging new flexible sensing technologies might be able to provide promising and effective solutions [39]. Recent advances in multimaterial direct-ink-writing 3-dimensional printing techniques have enabled the fabrication of customized flexible electrode arrays with excellent stability and a high signal-to-noise ratio [40], which is helpful for enhancing the wearability and comfort of HD-sEMG electrodes. Additionally, it is also crucial to develop miniaturized systems with sufficient computing power to acquire and process these signals, especially for integrated systems like prostheses.

## Conclusion

This study proposes a cwCST-image-driven model for hand gesture recognition using the decomposed cumulative spike trains of MUs. Compared with 2 RMS-based methods and a CST-based method, the proposed cwCST-CNN model achieves a higher recognition accuracy. Further, the analysis of the separability and consistency of cw-CST and RMS in feature space demonstrates the superiority of cwCST-CNN with a higher SI and a lower RI. The outcome reveals the distinct activation patterns of MUs underlying cwCST-images and their effectiveness for gesture recognition, providing a novel solution for HMI and promoting the development of neural interfacing.

## Data Availability

The data included in this study are available upon reasonable request by contact with the corresponding author.
